# Food allergy‐related social anxiety: Novel conceptualization of an important but overlooked construct

**DOI:** 10.1111/pai.70189

**Published:** 2025-09-01

**Authors:** Melissa L. Engel, Linda J. Herbert, Ashley Ramos, Ruchi S. Gupta, Christopher M. Warren

**Affiliations:** ^1^ Pritzker Department of Psychiatry and Behavioral Health Ann & Robert H. Lurie Children's Hospital of Chicago Chicago Illinois USA; ^2^ Center for Food Allergy and Asthma Research and Institute for Public Health and Medicine, Feinberg School of Medicine Northwestern University Evanston Illinois USA; ^3^ Children's National Hospital and George Washington University School of Medicine Washington District of Columbia USA; ^4^ University Hospitals Rainbow Babies and Children's Hospital and Case Western Reserve University School of Medicine Cleveland Ohio USA; ^5^ Advanced General Pediatrics and Primary Care Ann & Robert H. Lurie Children's Hospital of Chicago Chicago Illinois USA

**Keywords:** anxiety, food allergy, mental health, psychology, psychosocial impact, quality of life, social anxiety

## Abstract

Accumulating literature has highlighted the substantial psychosocial burden experienced by many young people with food allergy (FA), with growing attention to FA‐related anxiety. To date, research and clinical practice addressing FA‐related anxiety have focused specifically on understanding and alleviating the fear of allergic reaction, which can sometimes even lead to a specific phobia of anaphylaxis. Here, we propose an additional construct of *FA‐related social anxiety*, which we define as fear of social scrutiny or negative evaluation by others due to FA. In social situations involving food, patients high in FA‐related social anxiety often worry about judgment from others. For example, they may fear appearing different when eating alternative safe food, seeming demanding when asking to speak with a manager at a restaurant, or being perceived as difficult and unappreciative when declining offered but potentially unsafe food. We distinguish fear of social scrutiny from fear of anaphylaxis and illustrate how each fear may present in various social contexts across development. We offer a conceptual model of the broader construct of FA‐related anxiety, as well as behavioral conceptualizations of FA‐related social anxiety. When unrecognized or untreated, high levels of FA‐related social anxiety may lead to maladaptive social impacts (e.g., missed social opportunities) or adverse allergy outcomes (e.g., reluctance to carry epinephrine or engage in self‐advocacy, increasing propensity for allergic reaction). We discuss potential treatment implications, as well as the importance of future consideration of this construct in both research and clinical contexts.

**Key message**

Food allergy‐related social anxiety is highly salient and burdensome for many young people with food allergy (FA) yet is not captured by existing measures, which focus on fear of anaphylaxis. Consequently, it may often be unrecognized and untreated. Behaviorally, FA‐related social anxiety may manifest as avoidance of social situations involving food, along with avoidance of adaptive self‐management behaviors (e.g., carrying epinephrine auto‐injectors, checking ingredients, engaging in self‐advocacy), which may be perceived as likely to draw unwanted attention to FA. Ultimately, FA‐related social anxiety may contribute to psychological distress and thwarted social development, as well as suboptimal FA self‐management behaviors and outcomes.

Pediatric food allergy (FA) directly affects approximately 1 in 12 children in the United States and has increased in prevalence over recent decades.^1^, ^2^, ^3^, ^4^ Accumulating literature has highlighted that pediatric FA is costly not only due to its physical health effects and economic toll, but also due to the substantial psychosocial burden experienced by many young people with FA.^5^, ^6^, ^7^, ^8^, ^9^, ^10^, ^11^ Given the centrality of food to daily life, seemingly innocuous, enjoyable activities like eating lunch in the school cafeteria, attending birthday parties, or dining in restaurants may involve extensive planning and associated psychosocial stress. Research highlighting the psychosocial difficulties of living with FA has proliferated across the last two decades, perhaps jointly reflecting the increasing prevalence of FA and the growing recognition of general mental health challenges as a major public health issue. To date, the most common measures of FA‐related psychosocial difficulties have assessed health‐related quality of life, which may include social and emotional challenges.^8^, ^12^ More recently, anxiety has risen to the forefront of FA‐related mental health research and practice.

## 1 ANXIETY NOSOLOGY

Before examining FA‐related anxiety, we will orient the reader to the overall construct of anxiety and how it relates to FA by providing a brief overview of nosology. The Diagnostic and Statistical Manual of Mental Disorders, Fifth Edition (DSM‐5) classifies anxiety phenotypes into 11 distinct disorders.^13^ *Generalized anxiety* is often conceptualized as the prototype of anxiety and reflects excessive and difficult‐to‐control worry and anxiety about multiple aspects of daily life. For instance, an individual with generalized anxiety may worry about school or work performance, relationships, health and well‐being of loved ones, chores or household responsibilities, the weather, and/or current events. Anxiety screening questionnaires in research and clinical settings most commonly assess generalized anxiety. Findings regarding an elevated prevalence of such anxiety in young people with FA have been equivocal,^14^, ^15^ which is not necessarily surprising given that their worries may be isolated to FA‐specific experiences. In addition to generalized anxiety, another DSM‐5 anxiety diagnosis, *specific phobia*, is particularly relevant to FA. The DSM‐5 defines *specific phobia* as excessive, unreasonable fear in response to a specific context that leads to immediate anxiety, causes avoidance or significant distress, impairs daily functioning, and typically persists for at least 6 months. In efforts to capture the unique cognitions and behaviors that are often most burdensome for patients with FA, researchers have recently turned toward specifically examining “food allergy anxiety,” which is primarily described as a fear of allergic reactions that in some cases can constitute a specific phobia of anaphylaxis.^14^, ^16^, ^17^ Since this construct was proposed, progress has rapidly advanced to the creation of a measure (Scales of Food Allergy Anxiety; SOFAA) and development of targeted interventions.^18^, ^19^, ^20^ This is a remarkable advancement; however, our work as researchers and clinicians specializing in FA suggests that FA‐related anxiety is not limited to fear of anaphylaxis. *Social anxiety* is defined by the DSM‐5 as excessive worry about social situations involving potential scrutiny by others. Such feared social situations must almost always provoke excessive anxiety leading to significant avoidance, impairment, and/or distress, typically persisting for at least 6 months. Social anxiety is salient for many patients with FA yet inadequately captured by current patient‐reported outcome measures—despite its potential as a highly clinically relevant and addressable target for psychosocial interventions.

## 2 PROPOSING A NEW CONSTRUCT: FOOD ALLERGY‐RELATED SOCIAL ANXIETY

Synthesizing fragmented research on social challenges related to FA and years of our own research, clinical, and outreach experiences within the FA community suggests that social anxiety is a prominent difficulty in patients with FA. While social *anxiety* per se has not been formally acknowledged in the FA literature, it is well‐established that FA is associated with social difficulties.^8^, ^9^, ^10^, ^11^, ^12^ For instance, one of only six items on the Food Allergy Independent Measure specifically asks about the impact of FA on social life.^21^ Likewise, in a study to develop the Food Allergy Quality of Life‐Teen assessment tool that included 18 troublesome aspects of FA, adolescents identified feelings of being a burden to others and limitations on social activities (e.g., parties, eating with friends, making plans spontaneously) as the third and fourth most troublesome aspects of FA.^22^ FA‐related bullying and teasing is also quite common,^23^, ^24^, ^25^ which may further increase vulnerability for fear of social judgment.

Literature in other pediatric populations further emphasizes the social difficulties that youth with food‐related diseases may experience, including fears of judgment and disclosure, avoidance of self‐management behaviors, and increased risky behavior for purposes of peer acceptance. For example, interviews of diabetes‐specific worries in youth with type 1 diabetes identified a subtheme of social judgment, which was characterized by unwanted attention, feeling different, avoiding disclosure, and fear of negative evaluation in romantic settings.^26^ Likewise, in a study of young adults with celiac disease, the majority of participants described celiac as having a moderate to major impact on their dating life. Moreover, many endorsed discomfort explaining precautions to restaurant personnel, hesitation to kiss their partner, and engagement in riskier behaviors while on dates in order to avoid discomfort.^27^ In an exploratory study of attachment in adolescents and adults with FA, participants with FA reported heightened attachment avoidance, discomfort with close relationships, and relationships as secondary, underscoring the social impact of FA.^28^ In our own work with patients with FA, including the first author leading Food Allergy Research and Education (FARE)'s Teen Talks and Child Chats programs for the past several years,^29^, ^30^ we have been struck by a clear fear of judgment from others (e.g., friends, classmates, teachers, potential romantic partners, restaurant personnel, airline personnel) that appears to exert a substantial impact on many young people with FA. Even young people with high levels of diligence, knowledge, and confidence in their ability to avoid allergens, as well as experience in effectively identifying and treating anaphylaxis, may find the unpredictability of navigating social situations and encountering potential judgment to be particularly anxiety‐provoking. This socially challenging aspect of FA is a distinct and potentially powerful determinant of quality of life in many day‐to‐day contexts, yet this may often go underappreciated by those caring for patients with FA.

Adapting from definitions of general social anxiety,^19^ we define *FA‐related social anxiety* as fear of social scrutiny or negative evaluation by others due to FA. To be clinically significant, this fear must be excessive, persistent, and interfere with daily life by causing significant distress, impairment, and/or avoidance. In social situations involving food, patients high in FA‐related social anxiety often worry about judgment from others. For example, they may fear appearing different when eating alternative safe food, seeming demanding when asking to speak with a manager at a restaurant, or being perceived as difficult and unappreciative when declining offers of potentially unsafe food. Importantly, this fear may often be solely allergy‐specific, meaning that patients with FA‐related social anxiety may only experience distress and impairment in FA‐related social situations (e.g., a class party at which allergenic food is served, but not at a party where they are confident their allergens are not present). That being said, a subset of these youth may also experience broader social anxiety, such as when speaking in class, talking to adults, or approaching unfamiliar peers.

**FIGURE 1:**
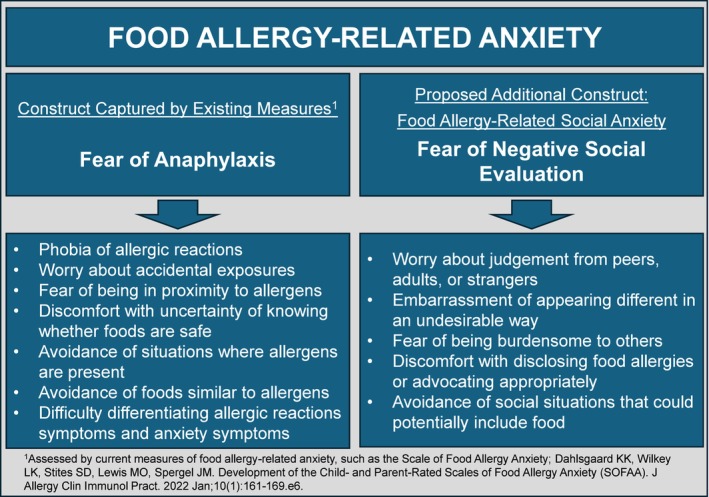
Overarching construct of food allergy‐related anxiety. Though not mutually exclusive, fear of anaphylaxis and fear of negative social evaluation are two distinct types of food allergy‐related anxiety.

Figure 1 illustrates how our proposed construct of FA‐related social anxiety differs from the more established FA‐related anxiety construct, fear of anaphylaxis, as well as how each subtype falls under the broader construct of FA‐related anxiety. The greater focus on fear of anaphylaxis versus fear of social scrutiny likely reflects the natural medical emphasis on physical safety versus social engagement. In a typical allergy visit, patients may be more likely to describe, and providers may be more apt to elicit, fear of allergen exposure, rather than exploring social impact. The emphasis on physical safety is understandable, yet addressing each type of anxiety is important for conceptualizing psychosocial burden, providing appropriate treatment, and promoting well‐being. Although not mutually exclusive, not indicative of diagnosable psychiatric disorders, and arguably adaptive at mild levels, these two types of FA anxiety are distinct and characterized by unique cognitions and behaviors that can be specifically targeted in psychosocial interventions.

Table 1 illustrates each clinical presentation during a variety of common social scenarios. As demonstrated, even in the same scenario, either (or both or neither) fear may be present. For example, two fictional patients, “Anaphylaxis Ali” and “Social Sam,” may have severe milk allergies and attend the same birthday party. Ali fears having an allergic reaction due to their proximity to allergen‐abundant pizza and cake. Although Ali knows they will not eat the provided party food, they feel fearful sitting at the same table as peers eating their allergen, worry about the possibility of contact reactions from touching shared surfaces, and hesitate to eat their own safe food due to the threat of anaphylaxis. In contrast, Social Sam fears negative social evaluation from other party goers. Although Sam feels confident that the homemade pizza and cake they packed are physically safe, they fear unwanted attention in the form of nonverbal gestures (e.g., staring from peers and employees), insensitive questions (e.g., “why are you eating that, does this look good to you, are you sure you don't want to try a bite?”) and unwelcome comments (e.g., “they're allergic to everything!”). These examples highlight that when assessing and treating FA‐related anxiety, it is critical that we understand not only the contexts that are likely to provoke FA‐related anxiety, but also the precise fear; only with this information can we tailor interventions appropriately.

**TABLE 1:** Examples of specific phobia of anaphylaxis and food allergy‐related social anxiety presenting in common social scenarios.

Scenario	Specific phobia of anaphylaxis^a^	Food allergy‐related social anxiety^a^
Eating Lunch in School Cafeteria	Fear of having an allergic reaction due to proximity to common allergens at table	Fear of being ostracized for eating different foods than everyone else, not eating at all, or sitting at a separate “allergy” table
Attending a Birthday Party	Fear of having an allergic reaction due to accidental exposure from undisclosed allergens	Fear of negative social evaluation for declining offered but unsafe food or bringing allergy‐safe food
Eating in a Restaurant	Fear of having an allergic reaction due to unsafe food preparation	Fear of speaking to a chef or manager to ask allergy‐related questions or request specific accommodations, including fear of becoming the “difficult customer” or “difficult table” at the restaurant
Traveling by Airplane	Fear of having an allergic reaction from being near allergens while not having easy access to emergency medical care	Fear of judgment from or negative interaction with the flight crew, airport personnel, or fellow airline passengers for engaging in allergy‐related precautions
Dating	Fear of having an allergic reaction from kissing	Fear of social rejection due to “ruining” the moment, consistently reduced spontaneity, and imposing food allergy‐related restrictions
Participating in a Catered Networking Dinner	Fear of having an allergic reaction due to unknown ingredients and high likelihood of cross‐contact	Fear of negative evaluation from professional colleagues, including potential employers, for appearing “difficult” by not eating or participating in some food‐oriented events, “demanding” by requesting a separate safe meal, or otherwise considered less positively than other colleagues due to need for appropriate accommodations

*Note*: In each social scenario, either or both of these fears may be present. When conceptualizing, assessing, and treating food allergy‐related anxiety, it is important to consider both the fear of anaphylaxis and the fear of social scrutiny.

^a^ In all examples, fears must be excessive, persistent, and cause significant distress or impairment (including avoidance) in order to be considered specific phobia of anaphylaxis or clinically significant food allergy‐related social anxiety. At mild levels, these fears may be expected and even adaptive, particularly if they are promoting self‐management behaviors.

## 3 BEHAVIORAL CONCEPTUALIZATION OF FA‐RELATED SOCIAL ANXIETY

**FIGURE 2:**
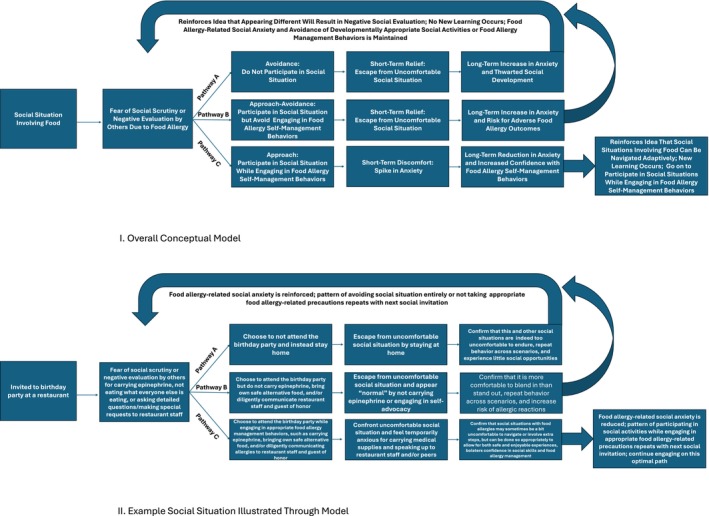
Behavioral conceptualization of food allergy‐related social anxiety. Model I illustrates the general proposed framework whereby social situations involving food elicit fear of negative evaluation. In such situations, patients with FA may avoid social participation, engage socially but avoid self‐management behaviors, or participate socially while engaging in appropriate self‐management behaviors. Model II applies a specific social scenario to this conceptual framework.

Figure 2 provides a behavioral conceptualization of FA‐related social anxiety, illustrating both our general model and an applied example. For patients with high levels of FA‐related social anxiety, any social situation involving food may trigger fear of social scrutiny due to FA. In such situations, patients may choose one of two maladaptive pathways, including (A) avoiding the social situation by not participating (e.g., stay home) or (B) participating in the social situation but avoiding engaging in FA self‐management behaviors (e.g., refrain from carrying epinephrine or communicating allergies to others). In general, anxiety is maintained by avoidance of feared stimuli.^30^, ^31^ In each of these pathways, patients with FA escape from uncomfortable social situations in the short term, which temporarily reduces their anxiety. Although avoidance provides relief in the moment, it increases anxiety in the long run and reinforces the belief that appearing different due to FA will result in negative social evaluation. Rather than learning that they can confront their fears by participating in social situations and engaging in appropriate FA self‐management behaviors, no new learning occurs, and the cycle of anxiety is instead strengthened or maintained.^31^, ^32^

When FA‐related social anxiety results in social avoidance (Pathway A), patients may miss out on social opportunities, potentially hindering the development of personal and professional relationships (e.g., family, friends, romantic partners, professional colleagues or potential employers). For example, young people may respectfully decline a birthday party invitation, believing that it is easier to avoid potential embarrassment and social awkwardness by just skipping the party entirely. When this pattern is repeated, the belief that attending the birthday party will result in negative social evaluation is reinforced. In other words, the fear of attending social gatherings and managing FA by bringing one's own food, not eating, speaking up, and/or garnering unwanted attention becomes even scarier. For adolescents and young adults, FA‐related social anxiety may manifest as avoiding dating due to fear of romantic rejection due to FA, as well as uncomfortable conversations surrounding FA‐related restrictions in the context of dining out, cooking, and kissing. Beyond social and romantic impacts, FA‐related social anxiety may also exert a substantial impact as young people enter the workforce. First‐time jobs for young people (e.g., restaurant server, fast food attendant, barista, babysitter, camp counselor) commonly involve handling potential allergens; young people with FA may experience social anxiety surrounding conversations with employers and colleagues about what foods they feel comfortable handling. Interviews and networking activities often involve food as well; for those with FA, trying to appear “normal” in such evaluative contexts may pose significant anxiety.

Although far more research is needed in this area, youth who experience FA‐related social anxiety may find it challenging to adopt developmentally appropriate levels of independence and self‐management. This may be especially pertinent when family members are highly involved in FA communication, such as caregivers routinely requesting restaurant accommodations rather than encouraging youth to self‐advocate. Recent guidelines have highlighted steps families and providers can take to ensure youth adaptively transition to FA self‐management.^33^, ^34^ Drawing from the larger anxiety literature,^31^, ^32^ it may be helpful to ensure caregivers are modeling and promoting confidence in young people's ability to participate, speak up for themselves, and manage FA in a wide variety of social situations.

Another maladaptive outcome of FA‐related social anxiety is engagement in risky FA behavior (Pathway B), whereby patients may exhibit thriving social lives yet fail to engage in important FA management behaviors due to fear of negative peer evaluation, thus heightening vulnerability to adverse allergy outcomes. For example, patients may refrain from checking ingredients, carrying epinephrine, or adequately communicating their needs, all of which may increase risk for anaphylaxis. Although FA‐related social anxiety may be detrimental to social functioning and physical safety at any age, it may be especially relevant during adolescence, a developmental period characterized by peer influence, risk‐taking, and identity formation,^35^, ^36^ and during which risk of anaphylactic fatalities may be particularly high.^37^, ^38^ Recent articles have highlighted the challenges of social pressure and peer influence among adolescents with FA,^23^, ^39^ with participants consistently reporting greater comfort engaging in FA management behaviors with family members than with peers, as well as substantial numbers engaging in increased risk‐taking behaviors when with peers. Rather than simply belabor the importance of reading labels, inquiring about cooking practices, educating peers, and carrying epinephrine, it may be worthwhile to address the FA‐related social anxiety that may underlie certain risky FA behaviors.

While Pathways A and B confer risk for maladaptive social and physical health outcomes, respectively, there fortunately exists a more adaptive third option. In Pathway C, patients may experience FA‐related social anxiety when confronted with a social situation involving food. However, these patients do not let their anxiety stop them from participating (Pathway A) or stop them from carrying their epinephrine, taking necessary precautions, and engaging in self‐advocacy (Pathway B). Rather, patients on Pathway C participate in social activities while engaging in appropriate FA self‐management. While they may experience a short‐term increase in anxiety, they experience long‐term gains in confidence and competence, fueling them to subsequently accept social experiences while also attending to their FA needs. Allergists, pediatricians, and caregivers can promote this more adaptive third path by acknowledging and normalizing the existence of FA‐related social anxiety yet equipping youth with confidence by modeling strategies for navigating social situations safely and smoothly. When FA‐related social anxiety is particularly distressing or impairing and prevents patients from pursuing this more adaptive approach, targeted psychosocial treatment may be warranted.

## 4 INTERVENTIONS FOR FA‐RELATED SOCIAL ANXIETY

Patients who experience FA‐related social anxiety may benefit from psychosocial interventions, such as cognitive behavioral therapy (CBT), specifically tailored to address the presenting fear. Just as allergists provide individualized FA management recommendations after targeted allergy assessment, psychosocial assessment and treatment should be tailored to each patient's unique concerns. In recent reviews, both of child anxiety at large^32^ and in phobia of anaphylaxis specifically,^40^ exposure and response prevention (ERP) has been identified as a key component of CBT that leads to decreased anxiety. However, ERP, which involves gradually confronting the feared stimuli in a safe environment, would look very different for fear of anaphylaxis versus fear of social scrutiny. As artfully illustrated by Dahlsgaard and colleagues,^39^ the former would include gradual proximity exposure to the allergen such as by looking at photos of a peanut, being in the same room as peanuts, eating at the same table as someone eating a peanut butter and jelly sandwich, smelling peanut butter, and perhaps touching a peanut prior to washing hands. On the other hand, the latter would include practicing noticeable FA management behaviors, such as visibly carrying epinephrine auto‐injectors, eating noticeably different foods in various social situations, calling restaurants to ask about ingredients or cross‐contact, and talking about FA with peers. As with CBT with ERP for anxiety at large, both in‐session exposure and home practice would likely promote greatest treatment gains. Given the salience of peers, group interventions for FA‐related social anxiety may be particularly effective.

## 5 FUTURE DIRECTIONS

Given the present dearth of work aiming to systematically describe, assess, and address FA‐related social anxiety, we encourage clinicians and researchers to (A) develop measures to assess this construct, (B) collect data to characterize this phenotype across representative samples, and (C) develop, implement, and evaluate resource‐efficient interventions that can specifically target FA‐related social anxiety in affected patients. The SOFAA was thoughtfully developed to quantify condition‐specific, observable behaviors indicative of fear of anaphylaxis, following realizations that generic anxiety measures may not capture the unique avoidance and behaviors experienced by patients with FA (see Dahlsgaard et al. (2022) for further discussion of limitations of broader anxiety measures). For similar reasons, we recommend construction of a brief, behaviorally based measure to assess FA‐related social anxiety. While general social anxiety is most common among adolescents and young adults, with age of onset most common in childhood and adolescence, age of onset and prevalence of FA‐related social anxiety remain unknown.^41^, ^42^ Further research is needed to identify the typical age of onset of FA‐related social anxiety and understand its developmental trajectory, as well as explore how this construct may present in caregivers of youth with FA. In the interim, providers can informally assess FA‐related social anxiety by asking patients and families open‐ended questions about their peer relationships, communication strategies, and self‐management behaviors in common social scenarios. For adolescents, these questions may include: “How does having FA impact your social life?”, “Are there any social situations you avoid due to FA?”, or “Do you ever find yourself worried about others judging you due to your FA? Tell me more about that.” For younger children, questions may include: “How comfortable do you feel telling your friends about your food allergies? What about adults” or “How do you feel when you are at a birthday party or on a class field trip and need to bring your own safe food?” Ideally, providers will engage in conversations with an eye toward crafting solutions that promote competent and confident FA management while also facilitating full participation in developmentally appropriate social activities and promoting overall psychosocial well‐being.

## AUTHOR CONTRIBUTIONS

**Melissa L. Engel:** Conceptualization; writing – original draft; writing – review and editing; visualization. **Linda J. Herbert:** Conceptualization; writing – review and editing; supervision; visualization. **Ashley Ramos:** Conceptualization; writing – review and editing. **Ruchi S. Gupta:** Resources; supervision. **Christopher M. Warren:** Writing – review and editing; supervision.

## FUNDING INFORMATION

None.

## CONFLICT OF INTEREST STATEMENT

Dr. Engel serves as Lead Programming Developer of Teen Outreach for Food Allergy Research and Education. Dr. Gupta receives research support from the National Institutes of Health (NIH) (R21 ID # AI135705, R01 ID # AI130348, U01 ID # AI138907), Food Allergy Research & Education (FARE), Melchiorre Family Foundation, Sunshine Charitable Foundation, The Walder Foundation, UnitedHealth Group, Thermo Fisher Scientific, Novartis, and Genentech. She serves as a medical consultant/advisor for Genentech, Novartis, Aimmune LLC, Allergenis LLC, and Food Allergy Research & Education (FARE). Dr. Gupta has ownership interest in Yobee Care, Inc. She is currently employed by Ann & Robert H. Lurie Children's Hospital of Chicago and is a Professor of Pediatrics & Medicine at Northwestern University Feinberg School of Medicine. We have no other potential conflicts of interest to disclose.

## PEER REVIEW

The peer review history for this article is available at https://www.webofscience.com/api/gateway/wos/peer‐review/10.1111/pai.70189.
